# Diagnostic Reliability of Ultrasound Compared to Magnetic Resonance Cholangiopancreatography and Endoscopic Retrograde Cholangiopancreatography in the Detection of Obstructive Jaundice: A Retrospective Medical Records Review

**DOI:** 10.7759/cureus.10987

**Published:** 2020-10-16

**Authors:** Sultan Alsaigh, Majed A Aldhubayb, Abdulaziz S Alobaid, Athkar H Alhajjaj, Bader A Alharbi, Deem M Alsudais, Hatoon A Alhothail, Mohammed A AlSaykhan

**Affiliations:** 1 General Surgery, King Fahad Specialist Hospital, Buraidah, SAU; 2 Radiology, King Fahad Specialist Hospital, Buraidah, SAU; 3 College of Medicine, Qassim University, Qassim, SAU

**Keywords:** endoscopic retrograde cholangiopancreatography, saudi arabia, sclerosing cholangitis, magnetic resonance cholangiopancreatography, obstructive jaundice

## Abstract

Background

Challenges in the diagnosis of obstructive jaundice include locating the level of obstruction, knowing the cause of obstruction, and differentiating between benign and malignant causes. Imaging plays a significant role in detecting the causes of obstruction. Radiologists aim to diagnose biliary obstruction, its level, extent, and probable causes to determine the appropriate treatment for each case.

Methods

Our study is a retrospective medical record review study. It included 150 patients who had ultrasound (US) diagnosis of biliary obstruction and underwent magnetic resonance cholangiopancreatography (MRCP) or endoscopic retrograde cholangiopancreatography (ERCP) in King Fahad Specialist Hospital, Buraidah. The patients’ medical records have been reviewed to measure the sensitivity and specificity of US, MRCP, and ERCP.

Results

Statistical analysis of the data showed that the sensitivity of US in detecting the most common cause of biliary obstruction, common bile duct (CBD) stone, was 26.6%, while the specificity was 100%. Comparing this sensitivity of US in detecting CBD stones to that of MRCP and ERCP, we obtained the following: US, 26.6%; MRCP, 62.9%; and ERCP, 62.4%. Although US was the least sensitive for detecting CBD stones, its specificity in this detection was 100%, while MRCP was 63.6%, and ERCP was 55.2%.

Conclusion

US is the best initial step for the diagnosis of biliary obstruction. However, MRCP and ERCP are more sensitive in detecting CBD stones compared to US. Also, compared to US, they have shown higher percentages in all aspects of detection: level, cause, and extent of biliary obstruction.

## Introduction

Although obstructive jaundice is a common problem, the diagnosis of biliary obstruction remains a challenge. The biggest challenges are locating the level of obstruction, knowing the cause of obstruction, and differentiating between benign and malignant causes.

The causes of biliary obstruction can be classified into benign and malignant. Benign biliary obstruction is the most common form of obstruction due to intra- and extrahepatic biliary stones, iatrogenic strictures, and inflammatory strictures due to sclerosing cholangitis or pancreatitis. Less frequently, benign biliary obstructions may include complicated choledochal cyst and Mirizzi syndrome. Malignant biliary obstructions are most caused by cholangiocarcinoma, pancreatic head cancer, gallbladder carcinoma, intrahepatic metastases, lymphoma, and metastatic lymphadenopathy [1].

A diagnosis of biliary obstruction can usually be established based on a patient’s medical history, physical examination, and blood test results. However, the location and cause of obstruction need further radiologic investigations. Obstructive jaundice in patients is a common indication for imaging, which has a major role in detecting the causes of obstruction. Radiologists aim to diagnose biliary obstruction: assessing its level, extent, and probable cause to determine appropriate treatment options for each patient. Ultrasound (US) imaging is considered the first-choice tool in diagnosing biliary obstructive diseases due to its accessibility, speed, ease of performance, and low-cost [2]. Moreover, it has proven to be sensitive in differentiating hepatocellular causes from other obstructive causes of jaundice in different studies [3]. US is recommended for an initial evaluation before using a more advanced noninvasive imaging option like magnetic resonance cholangiopancreatography (MRCP) or an invasive procedure like endoscopic retrograde cholangiopancreatography (ERCP) for further examination. Recently, MRCP has been used in detecting obstructive causes of jaundice, and it is more sensitive compared to US [4]. ERCP has been considered the gold standard for imaging biliary structure and the bile duct and pancreatic duct, and currently, ERCP has therapeutic applications [5,6].

ERCP is used for both diagnostic and therapeutic purposes for common bile duct (CBD) stone interventions. Also, ERCP has a complication rate of 5% to 10% and a mortality rate of 0.2% to 1.0% [7]. Unlike ERCP, MRCP is a noninvasive and rapid method that does not use ionizing radiation or contrast materials. Moreover, it helps assess biliopancreatic diseases and has indicated good results for detecting CBD stones [7-11]. When MRCP is not available, ERCP is used to assess biliary obstructions initially detected on US or CT. ERCP is the investigation of choice for suspected distal biliary obstruction that may require further interventions, such as sphincterotomy, basket retrieval of stones, biliary biopsy, or biliary stent placement [12].

Objectives

The objectives of this study were to test the sensitivity and specificity of US imaging modality in detecting obstructive jaundice, compare the sensitivity of US with that of ERCP and MRCP, and test the reliability of different imaging methods in detecting level and causes of biliary obstruction.

## Materials and methods

We conducted an analytical, retrospective review of patient medical records. All medical records of patients who had been diagnosed with obstructive jaundice on US from January 1, 2016, to March 31, 2019, in King Fahad Specialist Hospital (KFSH), Buraidah, which is the largest hospital in this area, were reviewed. Buraidah is the capital of Al-Qassim region in Saudi Arabia, which has a population of almost 600,000 people [13]. We included all adult patients who had US diagnosis of biliary obstruction and underwent MRCP or ERCP. Patients with active liver diseases or hemolysis were excluded from the study. Eventually, we included all 150 patients who met the criteria in this chosen period. We reviewed their medical records to evaluate and compare the results of US, MRCP, and ERCP imaging modalities. The study protocol was approved by the ethics committee of KFSH. The study was conducted in full accordance with the Declaration of Helsinki. The requirement for written informed consent was waived per the retrospective nature of the study.

Data collection

The patients’ medical data were retrospectively collected from their medical records. Then, we applied the inclusion and exclusion criteria. After that, we labeled the enrolled patients’ records numerically from one to 150 to keep the patients anonymous. All radiological reports were reviewed. Then, the collected data were entered into an Excel (Microsoft® Corp., Redmond, WA) file that was imported into a Statistical Package for Social Sciences (SPSS) (IBM Corp., Armonk, NY) file for statistical analysis to assess the sensitivity and specificity of each modality: US, MRCP, and ERCP

Statistical analysis

All data were analyzed using IBM SPSS Statistics for Windows, version 24.0 (IBM Corp., Armonk, NY). In addition, the Chi-square (X^2^) test was used for categorical variables, and the Mann-Whitney U test for continuous variables. P-value < 0.05 was considered statistically significant, with 95% confidence intervals.

Ethical approval and confidentiality

The data were collected anonymously and retain no identifying information. When the data were collected, we applied the inclusion and exclusion criteria. Then, the data were entered into an Excel file.

## Results

The sensitivity, specificity, positive, and negative predictive values of US, MRCP, and ERCP for detecting CBD stones, gallstones, and intrahepatic dilatation (IHD) are presented in Table 1.

**Table 1:** Main specificity and sensitivity results CBD, common bile duct; IHD, intrahepatic dilatation; US, ultrasound; MRCP, magnetic resonance cholangiopancreatography; ERCP, endoscopic retrograde cholangiopancreatography

	Diagnostic feature	Sensitivity (%)	Specificity (%)	Positive predictive value (%)	Negative predictive value (%)
US	CBD stone	26.6%	100%	100%	31.6%
	Gallstone	61.5%	32.4%	72.8%	22.2%
	IHD	49.5%	78.4%	87.1%	34.5%
MRCP	CBD stone	62.9%	63.6%	90.7%	23.3%
	Gallstone	60.5%	36.4%	84.3%	14.0%
	IHD	50%	59.1%	87.3%	17.0%
ERCP	CBD stone	62.4%	62.1%	86.9%	29.0%
	Gallstone	35.9%	55.2%	76.4%	17.6%
	IHD	41.0%	89.7%	94.1%	27.4%

Table 2 presents the distribution of biliary obstruction causes as detected by US, MRCP, and ERCP. Abnormal CBD diameter was noted in 109 cases (74.7%) on US, 124 cases (84.9%) on MRCP, and 117 cases (80.1%) on ERCP. CBD stone was seen in 29 cases (19.1%) on US, 86 cases (58.9%) on MRCP, and 84 cases (57.5%) on ERCP. Gallstone was seen in 92 cases (63.0%) on US, 89 cases (61.0%) on MRCP, and 55 cases (37.7%) on ERCP. IHD was seen in 62 cases (42.5%) on US, 71 cases (48.6%) on MRCP, and 51 cases (34.9%) on ERCP. In the detection of obstruction at different levels, the p-value was statistically significant for all causes except CBD stone.

**Table 2:** Distribution of cases of biliary obstruction * X^2^ is significant at 0.05. CBD, common bile duct; IHD, intrahepatic dilatation; US, ultrasound; MRCP, magnetic resonance cholangiopancreatography; ERCP, endoscopic retrograde cholangiopancreatography

Level	US (n=146)	MRCP (n=146)	ERCP (n=146)	X^2^	P-value
Diameter	109 (74.7%)	124 (84.9%)	117 (80.1%)	5.538	0.019*
CBD stone	29 (19.1%)	86 (58.9%)	84 (57.5%)	2.728	0.099
Gallstone	92 (63.0%)	89 (61.0%)	55 (37.7%)	8.949	0.000*
IHD	62 (42.5%)	71 (48.6%)	51 (34.9%)	5.265	0.022*

Table 3 shows that most patients underwent ERCP and that multiple procedures such as sphincterotomy and stone extraction have been performed in 30.8% of the patients. In our study, 23.3% of patients underwent sphincterotomy; 14.4% underwent sphincterotomy and plastic stent; 5.5% received sphincterotomy and metallic stent; 4.2% received sphincterotomy, stone extraction, and plastic stent. The patients who underwent metallic stent, stone extraction, or sphincterotomy, stone extraction, and metallic stent, represented 0.7% in each type of procedure equally. However, 17.8% of patients did not undergo any procedure at all.

**Table 3:** The procedure that patients underwent in endoscopic retrograde cholangiopancreatography

Procedure	Frequency	Percent
Metallic stent	1	0.7%
Plastic stent	3	2.1%
Sphincterotomy	34	23.3%
Sphincterotomy and metallic stent	8	5.5%
Sphincterotomy and plastic stent	21	14.4%
Sphincterotomy, stone extraction, and plastic stent	6	4.2%
Sphincterotomy and stone extraction	45	30.8%
Sphincterotomy, stone extraction, and metallic stent	1	0.7%
Stone extraction	1	0.7%
No Procedure	26	17.8%
Total	146	100%

Sphincterotomy was the most common procedure performed in 87% of patients, stone extraction in 36%, plastic stent in 20.5% of patients, and metallic stent in 6.8% of patients in our study.

## Discussion

The statistical analysis of our study showed that the sensitivity of US in detecting the most common cause of biliary obstruction (CBD stone) was 26.6%, while its specificity was 100%. When we compared the sensitivity of US in detecting CBD stone to that of MRCP and ERCP, the results were: US, 26,6%; MRCP, 62.9%; ERCP, 62.4%. Although ERCP is the gold standard, it's an invasive procedure with a higher possibility of complications than other imaging methods and should be used with caution. These results clearly explain a common approach of managing patients presenting with signs and symptoms of obstructive jaundice. After a thorough history and clinical examination, US might represent an initial radiologic investigation method. However, causes, level, and extent of biliary obstruction need more sensitive radiological interventions. Other parameters that can be detected using imaging modalities are shown in Tables 1, 2. All these modalities aid in and confirm the diagnosis of biliary obstruction. 

In a descriptive case study of 200 patients with cholestatic jaundice, US was able correctly to diagnose obstructions due to CBD stones (72.5%), dilated CBD without reason (41.7%), proximal obstruction (63.15%), distal CBD obstruction (60%), and sludge (66.7%). Accordingly, the overall ability of US in accurately diagnosing obstructive causes was 64.17% [14].

In a retrospective study of 413 patients with gallstones who underwent both US and MRCP, 26.39% of patients showed both gallstones and CBD stones. Just over half of all cases (55.05%) of CBD stones were diagnosed by US, while 44.95% of cases were not detected by US; they were confirmed by MRCP [15]. Another study compared the diagnostic value of US, MRCP, and ERCP in CBD stones in 384 patients and showed a diagnostic accuracy of US, MRCP, and ERCP to be 70.3% (260/370), 96.2% (356/370), and 100%, respectively [16].

In our study, while US was the least sensitive for detecting CBD stone, its specificity in detecting CBD stone was 100%. The specificity for MRCP was 63.6%, and ERCP’s specificity was 55.2%. Miao et al., in 2008, concluded that “the diagnostic accuracy of US for common duct stone was little higher, US should be used as a primary checking method.” However, Miao et al. did not compare sensitivity and specificity between imaging modalities, and they concluded that “combination of MRCP and ERCP can improve the diagnostic accuracy of common duct stone” [16].

Karki et al. reported a higher sensitivity of US for detecting CBD stone at 100%, which was higher than our finding of 26.6%. Karki et al. also reported US had a specificity of 89% for CBD stone detection, which was lower than our finding of 100%. Karki et al. concluded that due to its easy availability, noninvasive nature, and cost-effectiveness, US could be considered the first-line imaging tool [17]. Reports from both Karki et al. and Miao et al. accord with our results: US, MRCP, and ERCP are all reliable in detecting biliary obstruction, and by these given results, a consensus on US as an initial step investigation can be reached [16,17].

## Conclusions

We conducted this study to test the sensitivity and specificity of US in detecting obstructive jaundice, compare the sensitivity of US with that of ERCP and MRCP, and test the reliability of different imaging methods in detecting the level and causes of biliary obstruction. US is the best initial step for the diagnosis of biliary obstruction. However, MRCP and ERCP are more sensitive in detecting CBD stone compared to US, in which they have shown higher percentages in all aspects of detecting level, cause, extent of biliary obstruction compared to US.

## References

[1] Suthar M, Purohit S, Bhargav V, Goyal P (2015). Role of MRCP in differentiation of benign and malignant causes of biliary obstruction. J Clin Diagn Res.

[2] Kiani AA, Ghaffar A, Javaid RH, Khan S (2012). Ultrasonography in obstructive jaundice. Professional Med J.

[3] Chu JM, Cosgrove DO, McCready VR (1978). Ultrasound tomography of the liver: noninvasive method of choice for the differential diagnosis of jaundice. Aust N Z J Med.

[4] Kondo S, Isayama H, Akahane M (2005). Detection of common bile duct stones: comparison between endoscopic ultrasonography, magnetic resonance cholangiography, and helical-computed-tomographic cholangiography. Eur J Radiol.

[5] Filippone A, Ambrosini R, Fuschi M, Marinelli T, Pinto D, Maggialetti A (2003). Clinical impact of MR cholangiopancreatography in patients with biliary disease. Radiol Med.

[6] Upadhyaya V, Upadhyaya DN, Ansari MA, Shukla VK (2006). Comparative assessment of imaging modalities in biliary obstruction. Indian J Radiol Imaging.

[7] Loperfido S, Angelini G, Benedetti G (1998). Major early complications from diagnostic and therapeutic ERCP: a prospective multicenter study. Gastrointest Endosc.

[8] Freeman ML, Nelson DB, Sherman S (1996). Complications of endoscopic biliary sphincterotomy. N Engl J Med.

[9] Masci E, Toti G, Mariani A (2001). Complications of diagnostic and therapeutic ERCP: a prospective multicenter study. Am J Gastroenterol.

[10] Sperlongano P, Pisaniello D, Del Viscovo L, De Falco M, Parmeggiani D, Piatto A, Parmeggiani U (2005). Efficacy of magnetic resonance cholangiopancreatography in detecting common bile duct lithiasis: our experience. Chir Ital.

[11] Chan YL, Chan AC, Lam WW (1996). Choledocholithiasis: comparison of MR cholangiography and endoscopic retrograde cholangiography. Radiology.

[12] Lisle D (2013). Imaging for Students Fourth Edition. https://books.google.com.sa/books?id=-1hiAgAAQBAJ&lpg=PP1&ots=6wQR8s548l&dq=Imaging%20for%20Students%20Fourth%20Edition&lr&pg=PA69#v=onepage&q=Imaging%20for%20Students%20Fourth%20Edition&f=false.

[13] (2020). Saudi Arabia General Authority for Statistics. Sixteenth Service Guide 2017 Qassim Region. (Document in Arabic). General Authority for Statistics.

[14] Farrukh SZ, Siddiqui AR, Haqqi SA, Muhammad AJ, Dheddi AS, Khalid SK (2016). Comparison of ultrasound evaluation of patients of obstructive jaundice with endoscopic retrograde cholangio-pancreatography findings. J Ayub Med Coll Abbottabad.

[15] Qiu Y, Yang Z, Li Z, Zhang W, Xue D (2015). Is preoperative MRCP necessary for patients with gallstones? An analysis of the factors related to missed diagnosis of choledocholithiasis by preoperative ultrasound. BMC Gastroenterol.

[16] Miao L, Fan ZN, Ji GZ (2008). Comparative study of ultrasonography, magnetic resonance cholangiopancreatography and endoscopic retrograde cholangiopancreatography in common duct stones. (Article in Chinese). Zhonghua Wai Ke Za Zhi.

[17] Karki S, Joshi KS, Regmi S, Gurung RB, Malla B (2013). Role of ultrasound as compared with ERCP in patient with obstructive jaundice. Kathmandu Univ Med J (KUMJ).

